# Echocardiographic parameters during prolonged targeted temperature Management in out-of-hospital Cardiac Arrest Survivors to predict neurological outcome – a post-hoc analysis of the TTH48 trial

**DOI:** 10.1186/s13049-021-00849-7

**Published:** 2021-02-19

**Authors:** Thomas Hvid Jensen, Peter Juhl-Olsen, Bent Roni Ranghøj Nielsen, Johan Heiberg, Christophe Henri Valdemar Duez, Anni Nørgaard Jeppesen, Christian Alcaraz Frederiksen, Hans Kirkegaard, Anders Morten Grejs

**Affiliations:** 1 Department of Cardiology, Viborg Regional Hospital, Heibergs Alle 2K, 8800 Viborg, Denmark; 2 Department of Anaesthesiology and Intensive Care, Aarhus University Hospital, Aarhus, Denmark; 3 Department of Cardiology, Aarhus University Hospital, Aarhus, Denmark; 4 Centre of Head and Orthopaedics Rigshospitalet, Copenhagen, Denmark; 5 Department of Otorhinolaryngology, Head and Neck Surgery, Aarhus University Hospital, Aarhus, Denmark; 6 Department of Anaesthesiology, Regional Hospital West Jutland, Herning, Denmark; 7 Research Center for Emergency Medicine, Aarhus University Hospital, Aarhus, Denmark

**Keywords:** Cardiac arrest, Out-of-hospital cardiac arrest, Echocardiography, Targeted temperature management, Prognostication

## Abstract

**Background:**

Transthoracic echocardiographic (TTE) indices of myocardial function among survivors of out-of-hospital cardiac arrest (OHCA) have been related to neurological outcome; however, results are inconsistent. We hypothesized that changes in average peak systolic mitral annular velocity (s’) from 24 h (h) to 72 h following start of targeted temperature management (TTM) predict six-month neurological outcome in comatose OHCA survivors.

**Methods:**

We investigated the association between peak systolic velocity of the mitral plane (s’) and six-month neurological outcome in a population of 99 patients from a randomised controlled trial comparing TTM at 33 ± 1 °C for 24 h (h) (*n* = 47) vs. 48 h (*n* = 52) following OHCA (TTH48-trial). TTE was conducted at 24 h, 48 h, and 72 h after reaching target temperature. The primary outcome was 180 days neurological outcome assessed by Cerebral Performance Category score (CPC180) and the primary TTE outcome measure was s’. Secondary outcome measures were left ventricular ejection fraction (LVEF), global longitudinal strain (GLS), e’, E/e’ and tricuspid annular plane systolic excursion (TAPSE).

**Results:**

Across all three scan time points s’ was not associated with neurological outcome (ORs: 24 h: 1.0 (95%CI: 0.7–1.4, *p* = 0.98), 48 h: 1.13 (95%CI: 0.9–1.4, *p* = 0.34), 72 h: 1.04 (95%CI: 0.8–1.4, *p* = 0.76)). LVEF, GLS, E/e’, and TAPSE recorded on serial TTEs following OHCA were neither associated with nor did they predict CPC180. Estimated median e’ at 48 h following TTM was 5.74 cm/s (95%CI: 5.27–6.22) in patients with good outcome (CPC180 1–2) vs. 4.95 cm/s (95%CI: 4.37–5.54) in patients with poor outcome (CPC180 3–5) (*p* = 0.04).

**Conclusions:**

s’ assessed on serial TTEs in comatose survivors of OHCA treated with TTM was not associated with CPC180. Our findings suggest that serial TTEs in the early post-resuscitation phase during TTM do not aid the prognostication of neurological outcome following OHCA.

**Trial registration:**

NCT02066753. Registered 14 February 2014 – Retrospectively registered,

**Supplementary Information:**

The online version contains supplementary material available at 10.1186/s13049-021-00849-7.

## Introduction

Worldwide, out-of-hospital cardiac arrest (OHCA) remains a major challenge with estimated survival to hospital discharge of 7–10% [1, 2]. More than 87% of in-hospital deaths are due to brain injury or cardiac failure [3]. Following return of spontaneous circulation (ROSC), post-cardiac arrest syndrome and thus post-cardiac arrest myocardial dysfunction (PCAMD) contribute to cardiac failure and mortality [4]. PCAMD is characterised by global stunning of the ventricular function despite normal coronary blood flow [5, 6]. Ventricular dysfunction is present in the first hours (h) following ROSC and often normalises within the following 24-72 h [5, 7, 8]. Most previously studied transthoracic echocardiographic (TTE) indices such as left ventricular ejection fraction (LVEF) are prone to poor image quality and may overestimate left ventricular systolic function [9].

Targeted temperature management (TTM) for comatose survivors of cardiac arrest has been shown to improve survival and neurological outcome [10, 11] and is recommended by the International Liaison Committee on Resuscitation (ILCOR) as a post-cardiac arrest treatment for non-responsive survivors of OHCA [12]. The ﻿Targeted Temperature Management for 48 vs. 24 Hours and Neurologic Outcome After Out-of-Hospital Cardiac Arrest Trial (TTH48-trial) evaluated the effect of prolonged TTM at 33 °C for 48 h (TTM48) vs. 24 h (TTM24) following OHCA and found no difference in six-month neurological outcome or survival between groups [13]. In a sub-study from TTH48, we showed a significant beneficial effect of 33 °C TTM48 on left ventricular longitudinal myocardial function, assessed as peak systolic mitral annular velocity (s’), when compared with TTM24 [14].

The association between PCAMD, mortality and neurological outcome has been investigated in several studies, and reduced LVEF has been related to poor neurological outcome and mortality following OHCA [15–18]. Furthermore, some studies have suggested that an elevated E/e’ ratio is associated with in-hospital death and poor outcome following OHCA [19, 20]. However, results are inconsistent and existing studies lack methodological standardisation.

Our primary outcome Cerebral Performance Category (CPC) is a five-point scale with high inter-observer agreement recognized in the Utstein Guidelines as an optimal tool for reporting of neurological outcome following cardiac arrest [9, 21]. It is often dichotomized (CPC 1–2: good neurological outcome, CPC 3–5: poor neurological outcome) and widely used in previous studies of TTM in OHCA [10, 11, 13].

Acquiring optimal echocardiographic imaging in immobilized intensive care unit (ICU) patients undergoing TTM is challenging, and to accompany this challenge s’ was chosen as the primary echocardiographic measurement. s’ has proven to be a reproducible measure of left ventricular longitudinal myocardial function relatively insensible to poor image quality [22, 23]. Longitudinal fibres of the myocardium are situated in the sub endocardium making them prone to ischemic damage in case of significant coronary stenosis [24].

s’ is a strong predictor of mortality and adverse cardiovascular outcome following myocardial infarction independent of conventional echocardiographic findings [25, 26]. Amongst patients with known left ventricular systolic dysfunction, s’ is a predictor of cardiac death and hospitalization [27, 28]. However, whether changes in s’ following OHCA is prognostic of poor neurological outcome (CPC 3–5) has yet to be evaluated.

In the present study, we aimed to evaluate if early changes in systolic and diastolic myocardial function predict neurological outcome in comatose OHCA survivors treated with TTM. We hypothesised that change in s’ over time from 24 h to 72 h following start of TTM predicts the 180- day Cerebral Performance Category score (CPC180) [21] in comatose OHCA survivors treated with 33 °C TTM for 24 h or 48 h.

## Methods

### Study design

A sub-study of echocardiography data from 99 comatose OHCA survivors from the TTH48-trial was performed and the methods have been described in detail previously [13, 29]. In brief, the study was conducted according to the Declaration of Helsinki and approved by the Danish Health Research Ethics Committee (file number 20110022). After written informed consent from a legal next of kin and the patient’s general practitioner, patients were enrolled. Echocardiography data were obtained from 99 OHCA patients admitted to Aarhus University Hospital between February 2013 and July 2015.

The inclusion criteria were: comatose OHCA patients (Glasgow Coma Score (GCS) < 8) with suspected cardiac origin, age from 18 to 80 years, and continuous sustained ROSC for > 20 min.

Exclusion criteria were: > 60 min to ROSC, time from cardiac arrest to start of TTM > 4 h, terminal illness, coagulopathy, unwitnessed arrest with asystole as first rhythm, pregnancy, persistent cardiogenic shock (systolic blood pressure < 80 mmHg despite vasoactive treatment and/or aortic balloon pump intervention), CPC 3–4 prior to cardiac arrest, suspected intracerebral haemorrhage or stroke, acute coronary bypass grafting and/or lack of consent from a legal next of kin [22].

All prehospital data were collected using the Utstein Template [30]. According to the study protocol, all patients received 30 ml/kg isotonic saline at 4 °C prior to or at hospital admission.

At admission, significant coronary artery disease was diagnosed and treated according to guidelines, using emergency coronary angiography. In case of significant coronary artery disease, a percutaneous coronary intervention was performed.

### TTM intervention and randomisation

Patients were sedated using intravenous infusions of propofol and remifentanil/fentanyl and mechanically ventilated with a target Richmond Agitation-Sedation Scale score of − 5 [31]. Patients were cooled to a target temperature of 33 ± 1 C° using either endovascular (ICY® catheter, Thermogard XP, Zoll, US) or surface methods (Allon CureWrap®, CritiCool, MTRE, Israel). From a urinary catheter (Coviden™, Ireland), core temperature feedback was obtained to maintain target temperature. Cisatracurium was administered at TTM induction or if shivering occurred. Noradrenaline and/or inotropes were used if necessary to maintain mean arterial pressure > 60–65 mmHg. Patients were randomised online to TTM for 48 or 24 h using 1:1 randomisation with random block sizes within the first 23 h after admission. In the first 24 h, diuretics and crystalloids were administered to maintain a urinary output of > 1 ml/kg/h. After cessation of TTM therapy, rewarming was initiated at a rate of 0.5 °C/h.

### Echocardiography

Two-dimensional (2D) transthoracic echocardiography images were obtained for offline analyses. A Vivid E9 scanner equipped with a 1.5–4.5 MHz M5S phased array probe (GE Medical Systems Ultrasound, Norway) was used to record cine-loops at 24 h, 48 h, and 72 h after reaching the target temperature. Echocardiography outcomes were averaged from three cycles in sinus rhythm and five cycles in atrial fibrillation. The primary outcome measure s’ (average peak systolic mitral annular velocity) was obtained from colour tissue Doppler images (frame rate > 150 frames/sec). In order to diminish the weakness of s’ being insensible of global dysfunction, we improved our echocardiographic evaluation by scanning in three different left ventricular planes and measured mitral plane velocity in two regions of interest. s’ was averaged from six different positions around the mitral valve (septal, lateral, anterior, inferior, anterior-medial, and inferior-lateral). Global longitudinal strain (GLS) was obtained from 2D greyscale images using speckle tracking (frame rate 50–80 frames/sec) in the apical two-chamber, apical four-chamber, and apical long axis view. End of systole was defined as closure of the aortic valve. Biplane measurements were used to estimate LVEF with automatic speckle tracking (frame rate 50–80 frames/s). LVEF and GLS were used as secondary systolic outcome measures, and e’ and E/e’ were used as diastolic outcome measures [14]. Tricuspid annular plane systolic excursion (TAPSE) was used as outcome measure of right ventricular systolic function. Evaluation of the respiratory variability of the inferior vena cava (IVC) was applied as a surrogate marker of volume status. We used delta-IVC (ΔIVC) defined by maximum diameter of the IVC during expiration minus minimum diameter of the IVC during inspiration. A single investigator, blinded to randomisation and time of echocardiography, conducted all offline echocardiography parameter analyses using EchoPAC software BT 113 (GE Medical Systems Ultrasound, Norway).

### Neurological performance assessment

Follow-up was performed 180 days after enrolment. The primary outcome was CPC at 180 days (CPC180). CPC180 was assessed at a telephone interview by a researcher blinded to the results of randomisation and echocardiography. The outcome was dichotomised, and CPC180 scores of 1 (no neurological deficit) and 2 (mild to moderate dysfunction) were characterised as good neurological outcome, and CPC scores 3 (severe dysfunction), 4 (coma), and 5 (death) were characterised as poor neurological outcome. Neurological prognostication and decisions on withdrawal of treatment in the ICU was made by doctors independent of the group of investigators and the trial.

### Statistical analysis

Study population characteristics are presented as medians and interquartile ranges for continuous data and as absolute numbers and percentages for categorical values. The Student’s *t* test was used to compare continuous parametric values, the Chi-Square test for binary values, and the Mann-Whitney *U* test was used for non-parametric variables. This study is a post-hoc analysis of data from the TTH48-trial; power calculations have thus not been performed prior to patient inclusion.

A multiple logistic regression adjusted for primary rhythm status, time to ROSC, and age was performed to test if each of the systolic and diastolic outcome measures at 24 h, 48 h, and 72 h were associated with neurological outcome. To analyse the difference from 24 h to 72 h in the good and poor outcome group, a post hoc-analysis using linear combinations of estimators was performed if the model fitted the data set. Further, a repeated measures mixed model with unstructured covariance was used to calculate medians of each echocardiographic parameter and to estimate the differences between the good and poor outcome groups over time for each echocardiography parameter. Echocardiography data is presented as means (95% confidence intervals (CI)). Data was analysed using STATA 14 (StataCorp LP, TX, USA) and a *p*-value < 0.05 was considered statistically significant. Our analysis was a post-hoc sub analysis of a previously published randomised controlled trial with multiple continuous independent echocardiographic variables and thus correction of *p*-values for multiple testing was not applied.

## Results

### Study population and baseline characteristics

**Fig. 1:**
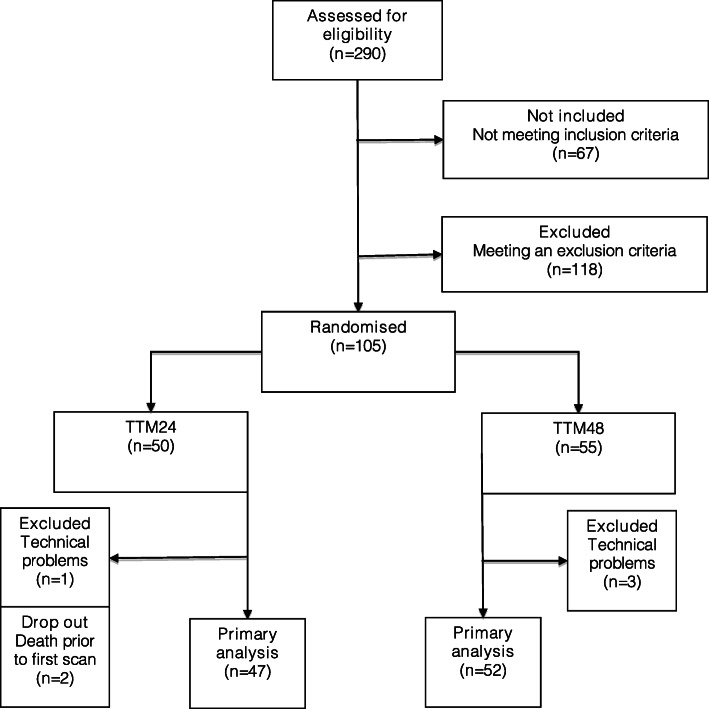
Consort diagram. Technical problems indicate problems with poor image quality where outcomes were not assessable

Ninety-nine patients were included in the final study population and the selection process is shown in Fig. 1.

The study population is presented in Table 1. Forty-seven patients (47%) were treated with TTM24 and 52 patients (53%) were treated with TTM48 (Table 1). In the TTM24 group the mean time from ROSC to extubation was 68.9 h (*n* = 32, 95%CI: 54.2–83.7), and in the TTM 48 group mean time from ROSC to extubation was 88.0 h (*n* = 34, 95%CI: 75.9–100.0, *p* = 0.05). At the 180-day follow-up across TTM-groups, 65 patients (66%) had a good neurological outcome and 34 patients (34%) had a poor neurological outcome (Table 1) with no difference between TTM groups. Patients with good outcome more often had a primary shockable rhythm (*p* = 0.03) and time from CA to ROSC was significantly shorter compared with patients with poor outcome (Table 1). Arrival lactate and Simplified Acute Physiology Score II (SAPSII) were significantly lower among patients with good outcome compared with patients with poor outcome (Table 1).

**Table 1:**
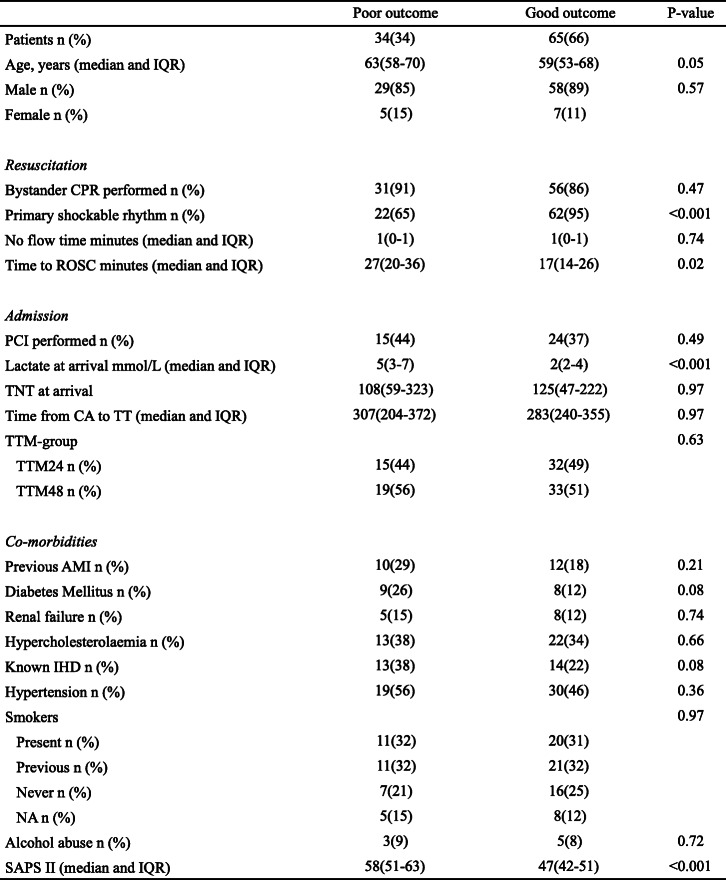
Baseline characteristics.

Data are presented as medians (interquartile range) for continuous data and absolute numbers (%) for binary data. *Abbreviations*: *AMI* Acute myocardial infarction, *CA* Cardiac arrest, *CPC* Cerebral performance category, *CPR* Cardiopulmonary resuscitation, *IHD* Ischemic heart disease, *IQR* Interquartile range, *NA* Not available, *PCI* Percutaneous coronary intervention, *ROSC* Return of spontaneous circulation, *SAPS* Simplified acute physiology score, *TNT* Troponin T, *TT* Start of therapeutic temperature management, *TTM* Therapeutic temperature management

### Left ventricular function

**Fig. 2:**
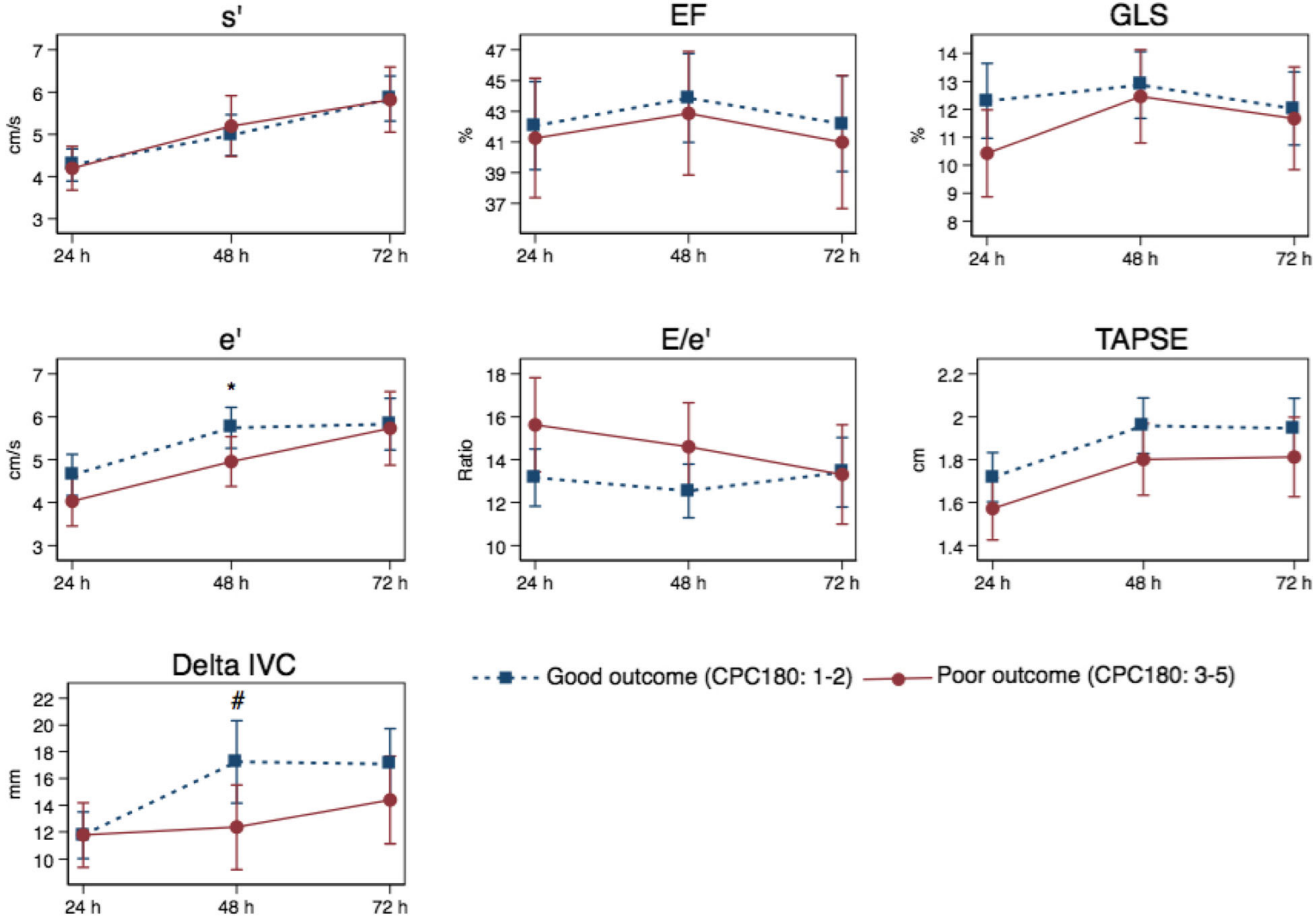
Estimated medians of echocardiographic outcomes. Echocardiography outcomes across all scan time points in the good outcome group and the poor outcome group, respectively. X-axes refer to scan time points. When testing for parallel curves, no graphs showed significantly different curves. Significant differences at time points are indicated below. Abbreviations: CPC180: cerebral performance category at 180 days, EF: ejection fraction, GLS: global longitudinal strain, h: hours, IVC: inferior vena cava variability, mm: millimetre, TAPSE: tricuspid annular plane systolic excursion. *: *p* = 0.044, #: *p* = 0.036

Neither our primary outcome s’ nor GLS and LVEF were associated with CPC180 across all three scan time points (Fig. 2, Additional file 1) or at each scan time point (Table 2, Fig. 2, Additional file 1). Estimated median e’ at 48 h was significantly higher in patients with good neurological outcome compared with patients with poor neurological outcome (*p* = 0.044) (Fig. 2, Additional file 1). However, when comparing changes over time, e’ was not significantly different between patients with good and bad outcome, respectively (Fig. 2, Additional file 1). E/e’ was not associated with neurological outcome neither at each time point nor across time points (Table 2, Fig. 2, Additional file 1). No parameter for left ventricular systolic or diastolic function showed significant differences when testing for parallel curves across all scan time points (Fig. 2, Additional file 1).

**Table 2:**
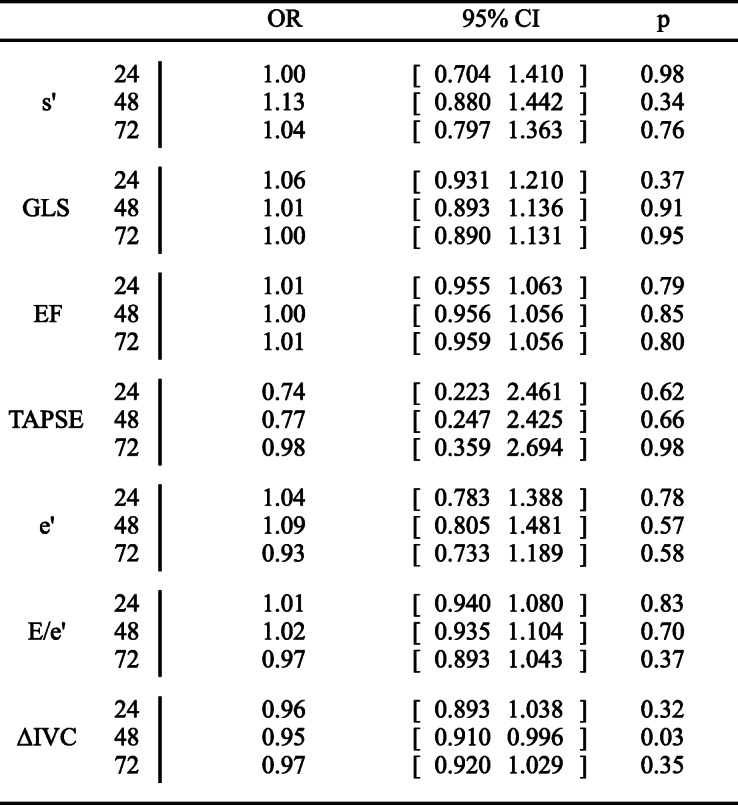
Echocardiographic indices and neurological outcome.

Multiple logistic regressions adjusted for primary rhythm, time to ROSC, and age. Higher OR depicts higher risk of poor outcome. The numbers 24, 48 and 72 to the right of the variable refer to echocardiography acquired 24 h, 48 h and 72 h following the start of targeted temperature management. *Abbreviations*: *EF* Ejection fraction, *GLS* Global longitudinal strain, *IVC* Inferior vena cava variability, *OR* Odds ratio, *TAPSE* Tricuspid annular plane systolic excursion, *95% CI* 95% confidence interval

### Right ventricular function

TAPSE was not associated with neurological outcome across any of the included parameters (Table 2, Fig. 2, Additional file 1); however, higher ΔIVC at 48 h predicted a poor neurological outcome (OR 0.95, 95%CI 0.910;0.996, *p* = 0.03) (Table 2). Furthermore, the estimated median ΔIVC at 48 h was significantly higher in patients with good outcome compared with patients with poor outcome (*p* = 0.036) (Fig. 2, Additional file 1). Neither TAPSE nor ΔIVC showed a significant result when testing for parallel curves across all scan time points (Fig. 2, Additional file 1).

## Discussion

This study showed no association between s’, global strain, LVEF, TAPSE, or E/e’ acquired after reaching the target temperature and 180 days neurological outcome. No echocardiography parameter showed consistent differences across all scan time points.

### TTE evaluation of cardiac function

In our study, e’ and ΔIVC at 48 h were the only TTE parameters associated with neurological outcome. The lower IVC variability found during hypothermia may reflect an elevated preload caused by diastolic dysfunction during hypothermia [32]. At 48 h, the TTM48 group was still hypothermic and the TTM24 group was normothermic adding to random variation at this point. At 72 h, more patients in the poor neurological outcome group were intubated and received positive pressure ventilation. Positive pressure ventilation is known to affect diastolic echocardiography parameters [33] and IVC dynamics [34]. Though not significant the duration of positive pressure ventilation was longer in the TTM48 group due to the prolonged cooling period compared with the TTM24 group. The prolonged positive pressure ventilation in the TTM48 group could be the reason for the lower e’ and ΔIVC at 48 h. However, these individual comparisons were not adjusted for the number of analyses for each echocardiography variable. As previously mentioned, correction of *p*-values was not performed, as it was not found suitable for this post-hoc analysis and not necessary to elucidate that the significant p-values in this study most likely represent type 1 errors. Only ΔIVC and e’ at 48 h turned out to be significantly different between the good and poor outcome group, but only at 48 h scan time and only ΔIVC was associated to neurological outcome at this scan time. However all p-values were remarkably close to 0.05 and the overall analyses for all echocardiography variables did not show any significant differences between groups and, hence as mentioned above, the significant differences at any individual time points are probably type 1 errors. A recent study found that low e’ was associated with in-hospital death following OHCA [19]. Significantly more patients with poor outcome were, however, intubated and thus the results may be confounded by the effect of positive pressure ventilation on diastolic function [19].

We previously showed a beneficial effect of TTM48 on s’ compared with TTM24; however, this effect was not seen across other echocardiography parameters [14]. Our study did not show an association between echocardiography measures of left ventricular systolic function and outcome. We included s’ and GLS in our evaluation of left ventricular systolic function since these markers are less preload-dependent and more sensitive to changes in left ventricular function compared with LVEF, especially following ischemia [23, 35–38]. In general, GLS may overestimate left ventricular function during ischaemia due to post-systolic shortening, adding to random variation [37]. However, as we defined the end of the systole as the closure of the aortic valve, post-systolic shortening should not have affected the GLS values included in this study. Heart rate increased during rewarming and may have affected speckle tracking and thus the GLS measurements [36].

Previously, we showed that s’ improved in patients treated with TTM48 [14] although TTM48 did not improve neurological outcome compared with TTM24 [13]. s’ was not associated with neurological outcome in our study, despite being highly reproducible and sensitive to changes in left ventricular systolic function and insensitive to poor image quality often encountered in intensive care patients [23, 39]. However, s’ is angle-dependent and measurements may be affected by respiration [23, 33].

### Timing of TTE and clinical implications

Our study evaluates the cooling and peri-rewarming phase during post-cardiac arrest TTM. PCAMD is present within the first hours following cardiac arrest and slowly recovers in the following days and months [4, 18]. LVEF, E/e’ and right ventricular diameter assessed following cardiac arrest have been shown to be associated with mortality and neurological outcome [15–18, 20, 40–42], though results are inconsistent [19, 43–45]. In studies showing an association between LVEF, E/e’, and right ventricular function TTE was performed within the first 24 h of admission to hospital [15–18, 20, 40–42]. In this period, the immediate effects of acute ischaemia of the heart and affected ventricular function are more pronounced [4, 18]. Patients in previous studies were heterogeneous and not comparable to our population with regard to lower rates of bystander CPR, longer times to ROSC, fewer patients had a primary shockable rhythm and survival rates were lower [15–18, 20, 40–42]. Patients in our study were immediately evaluated with coronary angiography and significant coronary lesions were treated. Thus, previous study populations have included more affected patients in the acute phase following cardiac ischaemia. Existing studies suggest that assessment of acute ischemia on left ventricular function has a greater potential of predicting neurological outcome compared with later scan times. However, the present study suggests that early serial TTE evaluation of cardiac function is not effective in prognosticating neurological outcome in the cooling and peri-rewarming phases following OHCA.

In our study, patients with good neurological outcome more often had a primary shockable rhythm, shorter times to ROSC, lower arrival lactate, and lower SAPSII scores (Table 1) indicating that prognostication of outcome following OHCA is a multimodal process including several clinical parameters [4]. Our data suggest that assessment of PCAMD on early serial TTEs following OHCA does not add important clinical value in prognostication of neurological outcome following cardiac arrest.

### Limitations

Our study has certain strengths as opposed to previous studies. We present results based on standardised early serial TTE scan times in a well-defined population of comatose OHCA patients. Standardised scan times were utilised to ensure comparability between data. Thus, our design enabled assessment of changes in TTE parameters over time and not only at single time point estimates. Few and experienced doctors performed all echocardiographies. One person blinded to patient treatment and outcome did the offline analyses, and inter- and intra-observer variability have previously been shown to be low [14]. Nonetheless, the process of patient inclusion in this study may have been subject to selection bias. Written consent was obtained from a legal next of kin following admission, which may have delayed randomisation slightly and, hence, the most ill patients may have been lost before study enrolment. As cardiac death often occurs early, this may have impacted on our results. In contrast to previous studies [19, 20], we excluded patients in prolonged cardiogenic shock, thus our selection process has probably favoured a population less ill compared to previous studies. Patients with good outcome more often had a primary shockable rhythm and were less likely to be diagnosed with diabetes (Table 1). Due to the number of patients in our analysis, we decided to adjust for a maximum of three variables (primary rhythm, time to ROSC and age). The rate of bystander CPR of 88% in our population was higher compared with previous studies [15, 19, 20, 46], but is comparable to the rate of bystander CPR reported in the TTM trial [47]. Only 34 patients had a poor neurological outcome limiting multivariate analysis and mixed model analysis in each group. Our analysis was a post-hoc analysis and power calculations have thus not been performed; type 2 error was a risk.

The scan time points in our study were standardised during cooling and rewarming. In an effort to limit missing data, three scan time points were chosen and thus, the TTM24 group had two scans during rewarming while the TTM48 group only had one. Comparing different rewarming periods may have added random variation to our results.

## Conclusion

Our study showed no consistent significant association between s’, GLS, LVEF, e’, E/e’, TAPSE or ΔIVC on early serial TTEs and 180-day neurological outcome in comatose survivors of OHCA. This study suggests that early serial TTE parameters in comatose survivors of OHCA are ineffective in predicting neurological outcome and do not add significant value to the already established prognostic process following OHCA.

## Supplementary Information

**Additional file 1:.** Estimated medians of each echocardiographic outcome measure at each scan time point.

## Data Availability

The datasets used and analysed during the current study are available from the corresponding author on reasonable request.

## Abbreviations

CPC180: Cerebral Performance Category
e’: Peak diastolic mitral valve annular velocity
E: Early diastolic mitral blood flow
TAPSE: Tricuspid annular plane systolic excursion
GCS: Glasgow coma score
GLS: Global longitudinal strain
h: Hours
ICU: Intensive care unit
ILCOR: International Liaison Committee on Resuscitation
IVC: Inferior vena cava variability
LVEF: Left ventricular ejection fraction
OHCA: Out-of-hospital cardiac arrest
PCAMD: Post-cardiac arrest myocardial dysfunction
ROSC: Return of spontaneous circulation
s’: Peak systolic mitral valve annular velocity
SAPSII: Simplified Acute Physiology Score II
TTE: Transthoracic echocardiography
TTH48: The ﻿Targeted Temperature Management for 48 vs. 24 Hours and Neurologic Outcome After Out-of-Hospital Cardiac Arrest Trial
TTM: Targeted temperature management
