# Supplementary material for: Echocardiographic parameters during prolonged targeted temperature Management in out-of-hospital Cardiac Arrest Survivors to predict neurological outcome – a post-hoc analysis of the TTH48 trial
Source: Scand J Trauma Resusc Emerg Med. 2021 Feb 19;29:37. doi: 10.1186/s13049-021-00849-7 (PMC7893899; doi:10.1186/s13049-021-00849-7)
Supplement: Supplementary file 1 — Additional file 1:. Estimated medians of each echocardiographic outcome measure at each scan time point. [file 13049_2021_849_MOESM1_ESM.doc]

**Additional file 1:**

**Supplementary 1:** Estimated medians of each echocardiographic outcome measure at each scan time point in the good outcome group and the poor outcome group. The numbers 24, 48 and 72 to the right of the variable name refer to echocardiographies acquired 24 hours, 48 hours and 72 hours following the start of targeted temperature management. Abbreviations: EF: ejection fraction, GLS: global longitudinal strain, IVC: inferior vena cava variability, TAPSE: tricuspid annular plane systolic excursion, 95% CI: 95% confidence interval.

*: Comparisons between medians at each scan time point

^#^: Overall analysis of effect over time
